# Supplementary material for: Efficient Physical Mixing of Small Amounts of Nanosilica Dispersion and Waterborne Polyurethane by Using Mild Stirring Conditions
Source: Polymers (Basel). 2022 Nov 25;14(23):5136. doi: 10.3390/polym14235136 (PMC9735813; doi:10.3390/polym14235136)
Supplement: Supplementary file 1 [file polymers-14-05136-s001.zip › polymers-2040435-supplementary.pdf]

Supplementary data

Efficient Physical Mixing of Small Amounts of Nanosilica Dispersion and Waterborne Polyurethane by Using Mild Stirring Conditions

María Echarri-Giacchi and José Miguel Martín-Martínez

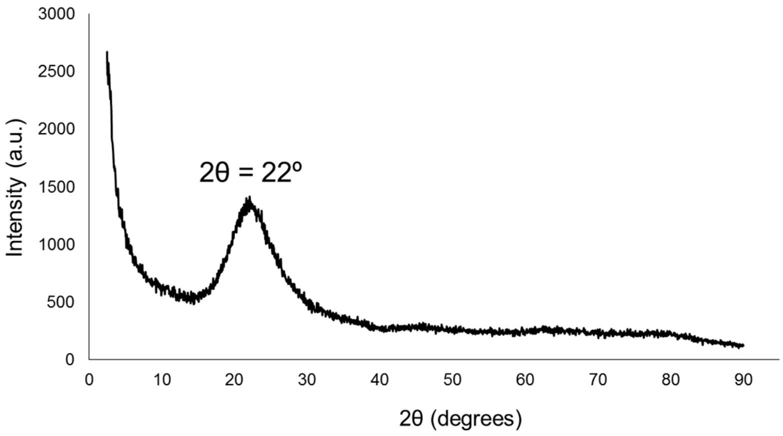

Figure S1. Wide angle X-ray diffractogram of the nanosilica powder.

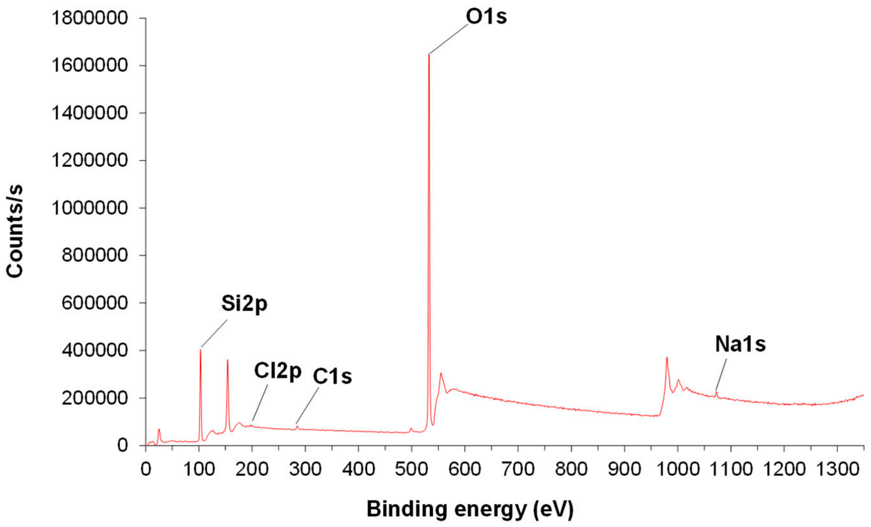

Figure S2. Survey XPS spectrum of the nanosilica powder surface.

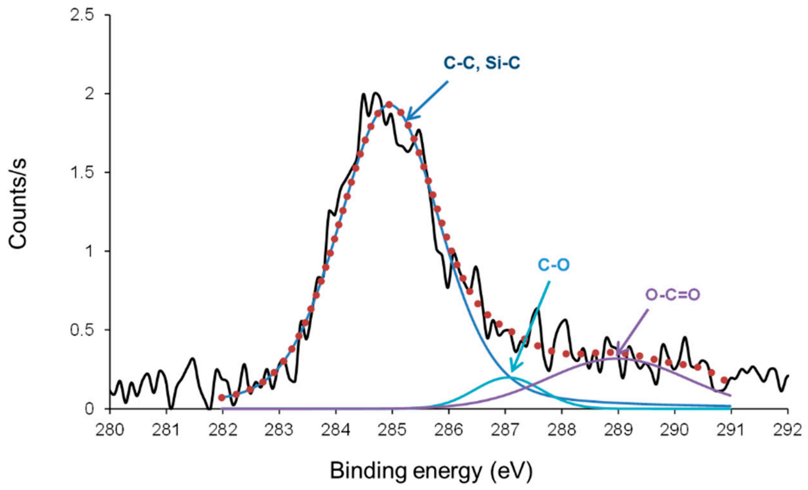

Figure S3. High resolution C1s spectra of the nanosilica powder surface. XPS experiment.

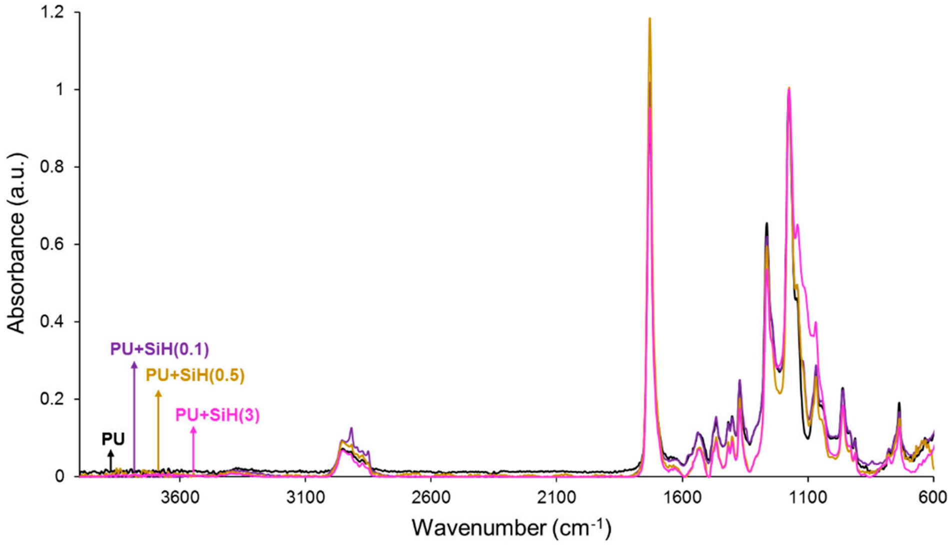

Figure S4. ATR-IR spectra of the PU and PU+nanosilica materials.

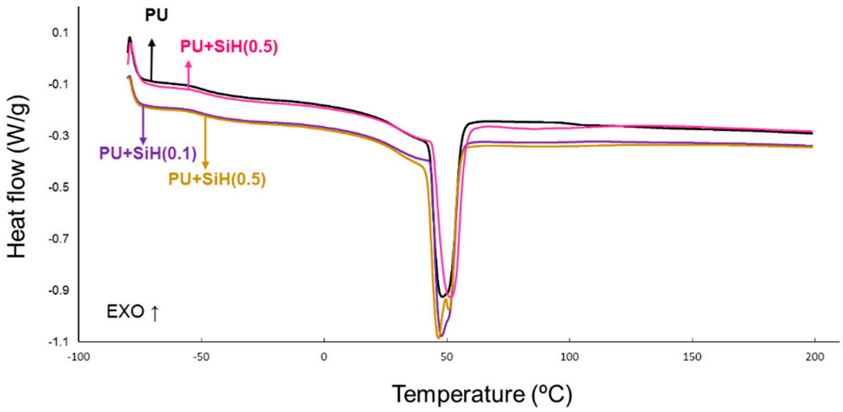

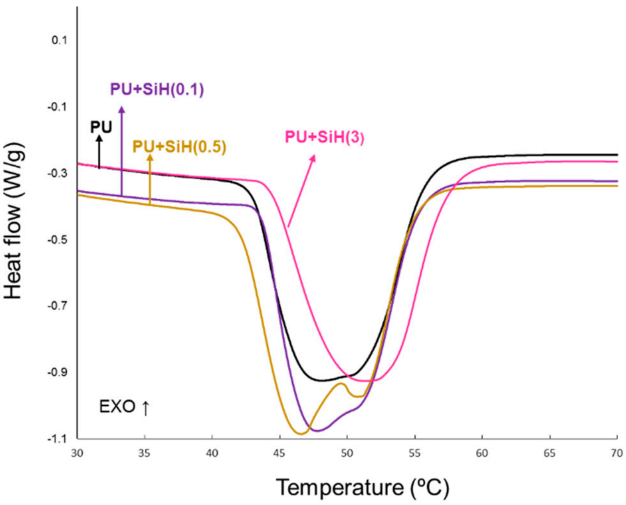

Figure S5. DSC curves of the PU and PU+nanosilica materials. First heating run. The figure below corresponds to the melting region of the DSC curves given above.

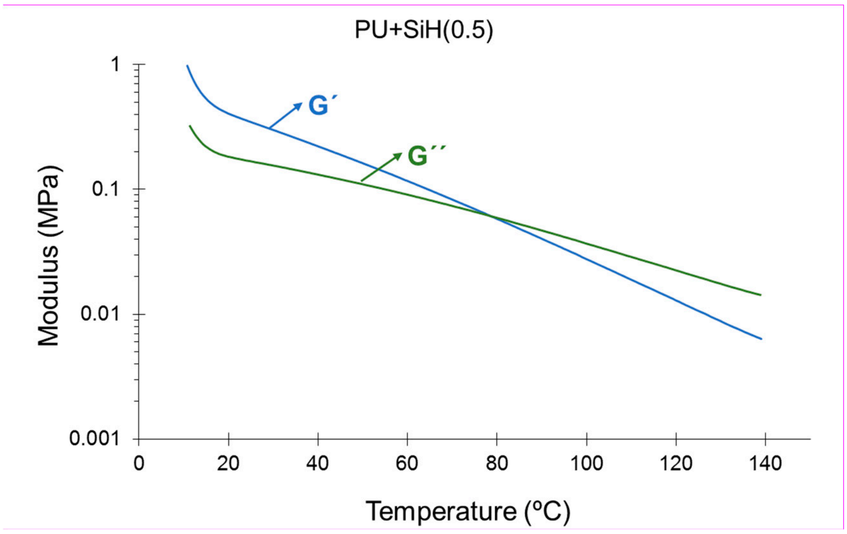

Figure S6. Variation of the storage (G´) and loss (G´´) moduli as a function of the temperature for PU+SiH(0.5).

Table S1. Mean particle sizes of the waterborne polyurethane dispersions without and with different amounts of nanosilica.

Dispersion	Particle size (nm)	Percentage (%)	Particle size (nm)	Percentage (%)	Particle size (nm)	Percentage (%)
Nanosilica	8	<0.5	140	12	768	88
PUD	212	67	253	22	297	11
PUD+SiH(0.1)	212	32	-	-	290	68
PUD+SiH(0.5)	202	46	265	54	-	-
PUD+SiH(3)	209	45	266	55	-	-

Table S2. Crystallization temperatures and enthalpies of the PU and PU+nanosilica materials. DSC curves. Cooling run.

Property	PU	PU+SiH(0.1)	PU+SiH(0.5)	PU+SiH(3)
T_c (°C)	-7	1	0	2
ΔH_c (J/g)	30	35	33	34

Table S3. Temperatures and weight losses of the thermal decompositions of the PU and PU+nanosilica materials. DTGA experiments.

	PU	PU+SiH(0.1)	PU+SiH(0.5)	PU+SiH(3)
T_1 (°C)	55	53	54	52
Weight loss$_1$ (%)	1	<1	<1	<1
T_2 (°C)	241	300	293	-
Weight loss$_2$ (%)	11	8	9	-
T_3 (°C)	329	324	325	356
Weight loss$_3$ (%)	66	12	22	42
T_4 (°C)	392	361	369	384
Weight loss$_4$ (%)	9	73	60	49
T_5 (°C)	436	441	440	442
Weight loss$_4$ (%)	5	4	4	3
